# Assessment of Catalase Inhibition Under e-Beam Irradiation

**DOI:** 10.3390/ijms26094358

**Published:** 2025-05-03

**Authors:** Victoria Ipatova, Ulyana Bliznyuk, Polina Borshchegovskaya, Alexander Chernyaev, Maria Toropygina, Violetta Kim, Alexander Nikitchenko, Aleksandr Kozlov, Dmitry Yurov, Mikhail Beklemishev, Igor Rodin, Elena Kozlova

**Affiliations:** 1 Skobeltsyn Institute of Nuclear Physics, Lomonosov Moscow State University, GSP-1, 1-2 Leninskiye Gory, 119991 Moscow, Russia; ipatova.vs15@physics.msu.ru (V.I.); alexeevapo@mail.ru (P.B.); a.p.chernyaev@yandex.ru (A.C.); d_yurov88@mail.ru (D.Y.); 2 Department of Physics, Lomonosov Moscow State University, GSP-1, 1-2 Leninskiye Gory, 119991 Moscow, Russia; ivantcova.vs20@physics.msu.ru (V.K.); nikitchenko.ad15@physics.msu.ru (A.N.); 3 Department of Medical and Biological Physics, I.M. Sechenov First Moscow State Medical University (Sechenov University), 8-2 Trubetskaya Str., 119991 Moscow, Russia; tim.mmit@yandex.ru (M.T.); fillnoise@mail.ru (A.K.); waterlake@mail.ru (E.K.); 4 Department of Chemistry, Lomonosov Moscow State University, GSP-1, 1-3 Leninskiye Gory, 119991 Moscow, Russia; beklem2@inbox.ru (M.B.); igorrodin@yandex.ru (I.R.); 5 Department of Analytical Chemistry, Lomonosov Institute of Fine Chemical Technologies, Moscow State Institute of Radiotechnics, Electronics and Automation—Russian Technological University (MIREA—RTU), 78 Vernadsky Avenue, 119571 Moscow, Russia

**Keywords:** catalase, accelerated electrons, radiation processing, radiation inhibition, spectrophotometry, oxygen bubbles, catalase activity, hydrogen peroxide, biodosimetry

## Abstract

Catalase serves as a crucial component of the antioxidant defense system by catalyzing the decomposition of hydrogen peroxide into water and molecular oxygen. This study investigated the effects of 1 MeV accelerated electron irradiation on catalase activity in model solutions at doses of 100 Gy and 1000 Gy. Enzyme activity was assessed using two complementary methods: spectrophotometric analysis and the oxygen bubble method. The experimental results demonstrated dose-dependent inhibition of catalase activity, indicating that substantial radiation-induced structural modifications may occur in the enzyme molecule as a result of irradiation. To understand the relationship between the irradiation dose and the catalase inhibition, calibration curves plotting the dependencies of hydrogen peroxide decomposition rate and the delayed appearance of oxygen bubbles after adding hydrogen peroxide to catalase saline solution on the catalase concentration showed a 1.5-fold reduction in catalase activity at 100 Gy and a 40-fold decrease at 1000 Gy. Based on these findings, we propose a novel biodosimetry approach utilizing the oxygen bubble formation delay time as an express assessment tool for detecting high radiation doses absorbed by biological objects, for example, food products. The results obtained in the study have important implications for evaluating radiation effects on biological systems, in particular catalase-containing food products, offering potential applications in radiation safety monitoring and food quality control.

## 1. Introduction

In the context of the global food crisis, one of the primary challenges is ensuring food safety and minimizing technological risks in the food industry [1]. Industrial e-beam irradiation has emerged as an effective tool for controlling pests, plant diseases, pathogenic microorganisms, and viruses in food products [2,3]. Electron beams have become increasingly used in the food industry since electron accelerators allow to vary the penetration depth of electrons and dose rate absorbed by food products by changing the operating mode and energy of electrons [4,5,6]. While high-energy electrons are used to treat the entire volume of animal products for full suppression of pathogens, low-energy electron beams are intended for antimicrobial treatment of plant and animal products and phytosanitary control of crops [7].

High food irradiation efficiency is achieved at optimal irradiation parameters, where target components—pathogenic and conditionally pathogenic microorganisms—are fully suppressed, while non-target components, such as proteins, including enzymes, lipids, carbohydrates, vitamins, and other high- and low-molecular-weight compounds, undergo minimal changes [3,8,9]. International standards regulate maximum irradiation doses to inhibit a wide range of pathogens [10]. However, as the variety of processed products is growing, it is essential to continuously refine the optimal dose ranges and establish quantifiable dose dependencies for different compounds, which can serve as indicators of functional and biochemical changes in non-target components in food product categories with diverse pathogens and different chemical composition [2,3,11,12].

The intensity of lipid and protein oxidation in foods affecting the overall quality of products during storage is regulated by antioxidants, such as vitamins, peptides, and enzymes, which neutralize radicals or scavenger metals in foods undergoing oxidation in the presence of oxygen [13]. Reactive oxygen species (ROS) formed in food products during storage as a result of natural biochemical processes, such as chain reactions of lipid oxidation, the action of oxidative enzymes, photooxidation, and bacterial and fungal activity, are attacked by enzymes [14,15,16]. Enzymes, including catalase, peroxidase, and superoxide dismutase, extend the shelf life of food products by regulating the intensity of oxidative processes in foods since they are responsible for the decomposition of ROS occurring in foods [17]. The lifespan of ROS is determined by the presence and composition of antioxidants, particularly enzyme catalase [18,19,20,21]. Catalase is a heme-containing enzyme composed of a polypeptide chain and four porphyrin heme (iron) groups. These heme groups enable the catalytic decomposition of H_2_O_2_ into water and oxygen. The activity of catalase, which is present in the vast majority of biological objects [20,21,22,23,24,25,26], can vary depending on different physical and chemical factors, such as temperature [19,27], medium acidity [19], storage time [28,29], vibration [30], chemical agents [22,23,31], electromagnetic fields [32,33], laser radiation [34], UV radiation [35], and gamma radiation [15], among others. There is evidence of the continued activity of catalase, which is present in different amounts in meat products of different type [36], after slaughtering during the entire period of storage [37].

Why is it important to study catalase activity after exposure to accelerated electrons? It is known that exposure to ionizing radiation affects both target and non-target components in food products, causing additional ROS to appear [8]. The combined action of ROS and irradiation intensifies lipid and protein oxidation, which has a negative impact on the organoleptic properties of foods, such as taste and smell. Since irradiation can damage non-target enzymes as well as other valuable molecules and reduces antioxidant content in food products, irradiation should be performed within an optimal dose range that does not lead to a significant inactivation of enzymes. The activity of catalase in an irradiated product can serve as a universally applicable marker of the damage to non-target enzymes due to irradiation since catalase is present in all plant and animal products [8].

Various methods for determining catalase activity are known, including spectrophotometric methods [20,22,24,38,39] and electrochemical methods [40,41]. Gasometric methods estimate catalase activity by measuring the volume of oxygen produced during the decomposition of H_2_O_2_. These include manometric methods [15] and foam height measurement [42]. Change in the catalase activity can serve as a quantitative indicator of the impact of irradiation on biological systems, particularly a wide range of food products.

Catalase activity is studied experimentally both directly in biological objects [20,23,25,28,38,41,42], and in model catalase solutions [15,19,24]. Since biological objects contain a wide range of antioxidants and enzymes, saline solution has been used to quantify the change in catalase activity after irradiation using the spectrophotometry method and the oxygen bubble method to avoid the interference of catalase with other antioxidants and enzymes. Catalase saline solution is irradiated with the doses typically applied to plant and animal products according to the food irradiation guidelines [4]. A clear dose dependency of catalase inactivation observed as a result of the action of low-energy electrons suggests that catalase activity can serve as a marker allowing to assess the extent of the damage of enzymes, which are essential for maintaining antioxidant system of food products during storage.

## 2. Results and Discussion

### 2.1. Research Stages

**Figure 1:**
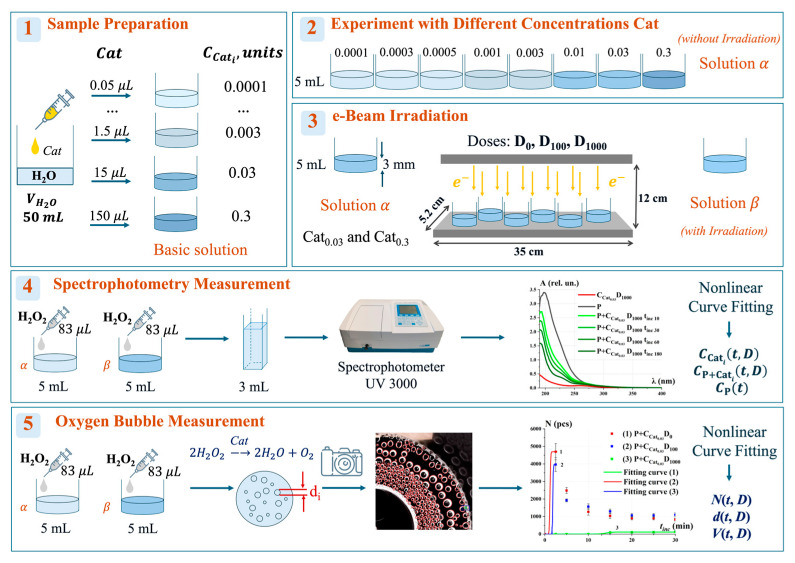
Stages of experimental studies: (**1**) preparation of catalase basic solution; (**2**) studies with different concentrations of catalase solution alpha (further referred to as *α*); (**3**) accelerated electron irradiation of solutions *α* and studies with irradiated solutions beta (further referred to as *β*); (**4**) spectrophotometry measurement of solutions *α* and *β*; (**5**) measurement of oxygen bubble formation in solutions *α* and *β*.

This study investigates the changes in catalase enzymatic activity following exposure to low-energy accelerated electrons with a maximum energy of 1 MeV at the doses of D_0_ = 0 Gy, D_100_ = 100 Gy, and D_1000_ = 1000 Gy. Figure 1 outlines the five stages of the study, with detailed descriptions of each stage provided in the Materials and Methods Section.

At the *first stage*, model saline solutions of catalase were prepared. The basic solution was made by adding different catalase enzyme (Cat) stock solution (Section 3.1) volumes (0.05–150 µL) to 50 mL of distilled water. To investigate the effect of catalase concentration on the catalytic reaction rate, non-irradiated solutions *α*, further referred to as “control solutions”, were prepared at concentrations ranging from Cat_0.0001_ to Cat_0.3_ units at the *second stage*.

At the *third stage*, model catalase solutions α with the concentrations of Cat_0.03_ and Cat_0.3_ were irradiated using the electron accelerator UERL-1-25-T-001 at the doses of D_0_ (non-irradiated), D_100_, and D_1000_ to obtain solutions *β*.

During *stage four* and *stage five*, solutions *α* and *β* were analyzed using two methods: spectrophotometry analysis using spectrophotometer UV 3000 and the oxygen bubble evaluation. For this purpose, 83 µL portions of 3% hydrogen peroxide (H_2_O_2_) were added to 5 mL of solutions *α* and *β*, initiating the catalytic reaction: 2H_2_O_2_ → 2H_2_O + O_2_.

### 2.2. Catalase Activity in e-Beam Irradiated Solutions β at Different Doses

#### 2.2.1. Catalase Activity in Irradiated Solutions *β*: Spectrophotometry Method

**Figure 2:**
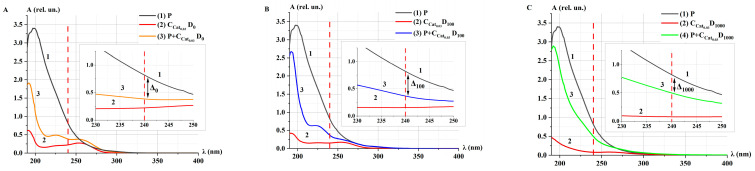
Absorption spectra of H_2_O_2_ (P, curve 1), catalase (Cat_0.03_, curve 2), and mix solution (P+Cat_0.03_, curve 3) after e-beam irradiation at different doses: (**A**) D_0_ (control), (**B**) D_100_, and (**C**) D_1000_. *t*_inc_ = 30 min; λ = 190–400 nm. The red dotted line marks the wavelength λ = 240 nm. The symbol ∆ and the black double headed arrow marks the difference in optical density (absorbances) between P and P+Cat_0.03_ at λ = 240 nm.

The spectrophotometric method was employed to analyze the enzymatic activity of model catalase solutions [19,22,24,43]. Figure 2 presents the absorption spectra of H_2_O_2_ solutions (P), model catalase solutions without the addition of peroxide (Cat), and catalase solutions with the addition of peroxide (P+Cat) after an incubation time (*t*_inc_) of 30 min. Each plot on the right side includes a zoomed-in scale for the wavelength range of λ = 230–250 nm.

After irradiation at the doses D_0_, D_100_, and D_1000_, the shape of the spectra of Cat_0.03_ (curve 2 in Figure 2) and P+Cat_0.03_ after *t*_inc_ = 30 min (curve 3 in Figure 2) changed. This may indicate the influence of accelerated electrons on the enzymatic activity of catalase in the decomposition of H_2_O_2_.

As a result of the catalytic reaction, a decrease in the optical density (absorbances) of P+Cat_0.03_ solutions at 240 nm was observed similar to other studies [19,22,24,43,44]. The difference (∆) in optical density of P and P+Cat solutions at 240 nm was greater for the control solution compared to the irradiated solutions: ∆_0_ > ∆_100_ > ∆_1000_. This indicates that the decomposition of peroxide by catalase is slower in irradiated catalase solution.

**Figure 3:**
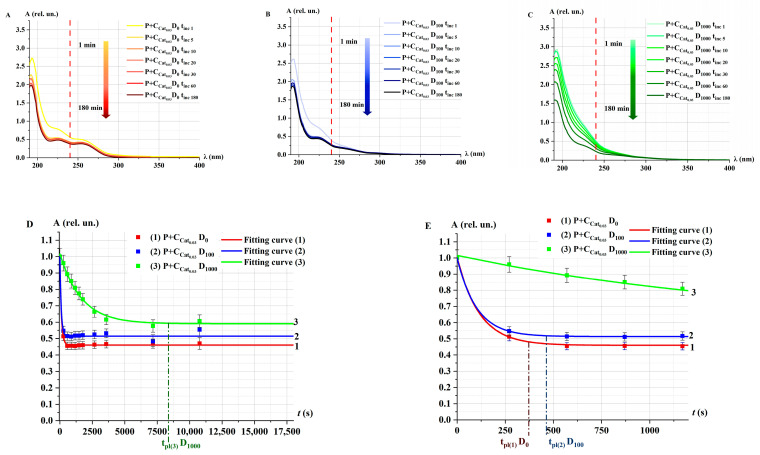
Absorption spectra of P+Cat_0.03_ solutions after accelerated electron irradiation at different doses: (**A**) D_0_ (control), (**B**) D_100_, and (**C**) D_1000_. *t*_inc_ = 1–180 min; λ = 190–400 nm. The red dotted line marks the wavelength λ = 240 nm. (**D**,**E**) Kinetic curves *A*(*t*); symbols represent experimental values (at λ = 240 nm). Fitting curves (Equation (1)) are represented by lines—D_0_ (curve 1), D_100_ (curve 2), and D_1000_ (curve 3). Normalization was performed on the value of optical density at *t*_inc_ = 0. (**D**) The measurements are presented in the range *t*_inc_ = 0–18,000 s (5 h); the time of reaching the plateau (*t_pl_*) is indicated by dotted lines (*t_pl_*_(3)_)D_1000_ for curve 3. (**E**) The measurements are presented in the range *t*_inc_ = 0–1200 s (20 min), and the times of reaching the plateaus are indicated by dotted lines (*t_pl_*_(1)_)D_0_ for curve 1, (*t_pl_*_(2)_)D_100_ for curve 2 (**E**).

Figure 3 shows the absorption spectra of the P+Cat_0.03_ solution after e-beam irradiation at the doses of D_0_, D_100_, and D_1000_ for different incubation times *t*_inc_.

After irradiation of model catalase solutions at the doses D_0_ and D_100_, an intensive decrease in the optical density of P+Cat_0.03_ solutions was observed during *t*_inc_ = 3–5 min. Upon further incubation up to 180 min, the rate of enzymatic decomposition of H_2_O_2_ remained unchanged (Figure 3A,B,D). In the case of catalase irradiated at the dose of D_1000_, the decrease in the absorption spectrum of the solution occurred throughout the entire incubation period (Figure 3C,D). Notably, even 3 h after the initiation of the catalytic reaction, the spectrum of the P+Cat_0.03_ solutions did not return to its original state—the spectrum of Cat_0.03_ (curve 2 in Figure 2A–C). This may be attributed to changes in the microstructure of the enzyme induced by irradiation.

The following function is proposed to describe the change of optical density of the P+Cat solution at a wavelength of 240 nm:

(1) $$A\left(t_{inc}\right)=A_{0}+B_{A}\cdot e^{-kt_{inc}},$$

(2) $$\upsilon_{A}=\left|A^{\prime }\left(t_{inc}=0\right)\right|=\left|-B_{A}\cdot k\right|,$$

where *A*_0_ and *B_A_* (rel. un.) are the initial optical density of undecomposed P and decomposed P, *k* (s^−1^) is the probability of interaction between Cat and P per unit time, and *υ_A_* (rel. un.) is the rate of catalytic decomposition of H_2_O_2_, representing catalase activity. For e-beam irradiated solutions P+Cat_0.03_, $\frac{\upsilon_{A}(D_{0})}{\upsilon_{A}(D_{100})}=1.5$, and $\frac{\upsilon_{A}(D_{0})}{\upsilon_{A}(D_{1000})}=40$.

In our experiments (Figure 3D), the optical density reached a saturation effect: *υ_A_* → 0 at *t*_inc_ → ∞. We consider that the plateau exit time is the time when the relative optical density is equal to 0.01$A_{0}$. In this case, $t_{pl}=-\frac{ln\left(\frac{0.01A_{0}}{B_{A}}\right)}{k}$. The corresponding time is indicated by the dotted lines in the Figure 3D,E. The following values $t_{pl}$ were established: $t_{pl(1)}$D_0_ = 370 ± 10 s, $t_{pl(2)}$D_100_ = 450 ± 11 s, and $t_{pl(3)}$D_1000_ = 8500 ± 50 s.

Similar plateau effects have been observed when catalase solutions were exposed to higher temperatures [27]. The mechanism of behind the plateau effect is not fully understood, but it may be related to catalase inhibition. It is known that high concentrations of H_2_O_2_ can alter the microstructure of the catalase enzyme, potentially leading to a decrease in its activity [22,40,45].

On the other hand, the distinct kinetics of optical density changes observed in Figure 3D can be attributed to the formation of oxygen bubbles in the P+Cat solutions. At higher catalase concentrations, the saturation effect increases the likelihood of oxygen bubble formation. These bubbles enhance the heterogeneity of the medium, reducing the probability of interaction between Cat and P molecules. As a result, the spectra show a slowdown in the decrease of H_2_O_2_ levels and a gradual plateau effect. It is important to note that diffusion processes are critical for catalase activity not only at the microlevel, such as oxygen bubble formation, but also at the molecular nanolevel [40].

**Figure 4:**
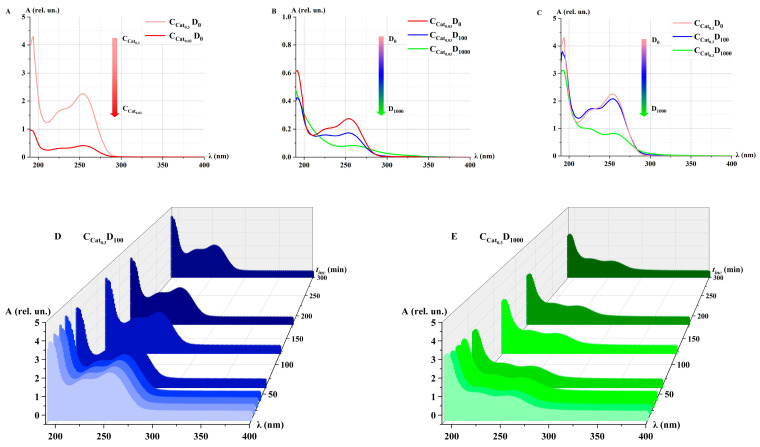
Absorption spectra of Cat model solutions after accelerated electron irradiation at different doses (*t*_inc_ = 10 min): (**A**) Cat_0.03_ and Cat_0.3_ at dose D_0_; (**B**) Cat_0.03_ at doses D_0_, D_100_, and D_1000_; (**C**) Cat_0.3_ at doses D_0_, D_100_, and D_1000_. Kinetics of changes in the absorption spectra of Cat_0.3_ model solutions at doses D_100_ (**D**) and D_1000_ (**E**). *t*_inc_ = 1–300 min; λ = 190–400 nm. The color of the absorption spectra of catalase model solutions from light tint to dark tint corresponds to increasing *t_inc_*. Blue colors—correspond to the dose of 100 Gy, green colors—1000 Gy.

Figure 4 shows the absorption spectra of Cat_0.03_ and Cat_0.3_ model catalase solutions after irradiation with the doses of D_0_, D_100_, and D_1000_.

The absorbance decreased for both concentrations of catalase (Figure 4A) and the doses of accelerated electron irradiation (Figure 4B,C). At the D_1000_ dose, a more intense decrease and change in the shape of the absorption spectrum of the catalase solution were observed. This radiation-induced effect, resulting from the action of accelerated electrons at different doses, may be attributed to changes in the conformation of the catalase molecule. A similar inactivation effect was also reported when gamma radiation was applied to an anhydrous sample of catalase [15].

After 3 h of irradiation with the doses D_100_ and D_1000_, no significant changes in the catalase spectrum were detected (Figure 4D,E). Based on this, it can be concluded that no structural and functional changes of the catalase were observed during 3 h after irradiation. Yet, a partial recovery of catalase damage was observed at 4 °C 48 h after gamma-irradiation [46]. Further research will monitor the catalase activity within a longer time frame to estimate the probability of catalase recovery after e-beam irradiation.

#### 2.2.2. Catalase Activity in Irradiated Solutions *β*: Oxygen Bubble Method

**Figure 5:**
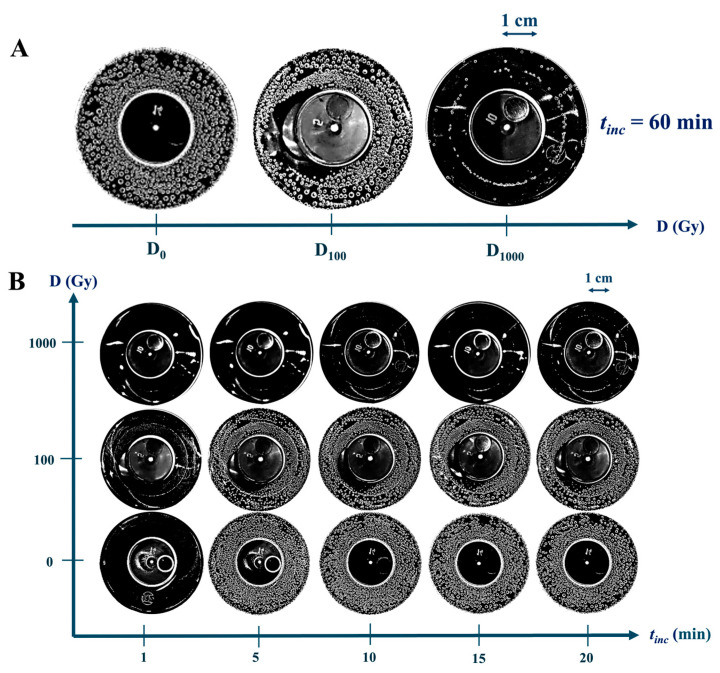
Photographs of oxygen bubbles in P+Cat_0.03_ solutions after e-beam irradiation at doses of D_0_, D_100_, and D_1000_. (**A**) *t*_inc_ = 60 min; (**B**) *t*_inc_ = 1–20 min.

Figure 5 shows photographs of P+Cat solutions with oxygen bubbles formed on the surface of solutions after exposure to accelerated electrons at different doses: D_0_, D_100_, and D_1000_.

In P+Cat_0.03_ solutions, where the Cat_0.03_ was irradiated at doses D_0_ and D_100_, abundant bubble formation was observed after *t*_inc_ = 60 min. The bubbles uniformly covered the bottom of the beaker, indicating high catalytic activity of the non-irradiated enzyme (Figure 5A). In contrast, samples with catalase irradiated at the dose of D_1000_ showed a significant decrease in both the number and diameter of bubbles. This suggests a substantial reduction in the catalytic activity of the enzyme following e-beam irradiation.

The kinetic variation of bubble formation in P+Cat_0.03_ solutions revealed the following: in the samples with catalase solutions irradiated at doses D_0_ and D_100_, intensive bubble formation was observed within the first 5 min. In contrast, for the D_1000_ dose, bubbles began to form in small amounts only after 13–15 min. This demonstrates a significant reduction in catalase activity at the D_1000_ dose.

**Figure 6:**
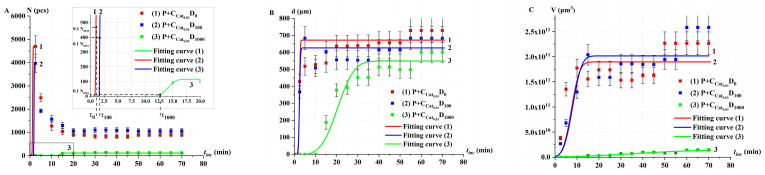
Kinetics of changes in oxygen bubble parameters in P+Cat_0.03_ solutions after accelerated electron irradiation at doses D_0_ (curve 1), D_100_ (curve 2), and D_1000_ (curve 3). (**A**) Number of bubbles *N*(*t*_inc_), (**B**) average diameter of bubbles *d*(*t*_inc_), (**C**) total volume of bubbles *V*(*t*_inc_). Dots represent experimental values (with error symbols), and curves show fitting curve (Equation (3)). The dotted lines indicate the delay time τ.

Figure 6 shows the kinetics of the changes in the number of oxygen bubbles *N*, their average diameter *d*, and the total volume *V* of all bubbles in P+Cat_0.03_ solutions after irradiation at doses D_0_, D_100_, and D_1000_. These parameters were calculated using the ImageJ program toolbox.

In Cat_0.03_ solutions irradiated at doses D_0_ and D_100_, an intensive increase in the number of oxygen bubbles was observed after adding P through *t*_inc_ = 2–3 min. However, with further monitoring, the number of bubbles decreased due to bursting and merging (Figure 6A). In contrast, for model catalase solutions irradiated at the dose of D_1000_, no bubbles were observed during the first 10 min. At *t*_inc_ = 15 min, bubbles began to nucleate, and their number eventually reached a stationary level. The observed effects of oxygen bubble emergence, growth, and stabilization align well with the foam formation phenomena described in [42]. Notably, no additional reagents were used to stop the reaction. The average diameter and volume of the bubbles showed a nonlinear increase with incubation time, eventually reaching saturation (Figure 6B,C).

The process of bubble formation exhibits a threshold effect based on the concentration of oxygen generated during the catalytic reaction. This agrees well with the data presented in [47], which demonstrate nonlinear effects of bubble formation as a function of gas concentration. To describe the changes in oxygen bubble parameters *N*, *d*, and *V* from *t*_inc_, the following function is proposed:

(3) $$F\left(t_{inc}\right)=\frac{B_{F}}{\sigma_{F}\sqrt{2\pi }}\int_{-\infty }^{t_{inc}}e^{\left(-\frac{{\left(t-t_{F}\right)}^{2}}{2{\sigma_{F}}^{2}}\right)}dt,$$

where *B_F_* = *B_N_* (pcs), *B_d_* (µm), and *B_V_* (µm^3^) represents the amplitude (maximum value of the corresponding parameter); *σ_F_* = *σ_N_* (min), *σ_d_* (min), and *σ_V_* (min) estimates the characteristic time of the oxygen bubble formation process; *t_F_* = *t_N_* (min), *t_d_* (min), and *t_V_* (min) represents the mathematical expectation of the characteristic time of bubble development. These parameters can be used to estimate the enzymatic activity of irradiated catalase.

Figure 5 demonstrates that oxygen bubbles appeared after a certain delay. In this work, the time of their appearance was determined based on the distribution curves (Equation (3)) plotted in Figure 6A. The delay time (τ), defined as the time required for 10% of the maximum number of bubbles (0.1 Nmax) to form under the given conditions, was chosen as the optimal parameter for rapid diagnosis of the enzymatic activity of catalase after irradiation.

The graphs in Figure 6 show that irradiation of model catalase solutions at a dose of 1000 Gy significantly reduces the rate of oxygen bubble formation as well as the total number and volume of oxygen bubbles. This suggests substantial damage to catalase molecules and a corresponding decrease in its enzymatic activity.

In these experiments, a model solution of catalase was used to evaluate its activity after exposure to an accelerated electron beam. Similar scientific works [15,46] also used model solutions of catalase to study the effect of irradiation on the enzyme activity. As was found in [15], gamma irradiation generated by radionuclide ^60^Co decreased catalase activity with an increase in the dose rate from 5.5 to 70 Gy/h. In our experiments, we detected a significantly delayed appearance of oxygen bubbles after adding hydrogen peroxide to catalase saline solution irradiated with the dose of 1000 Gy at a higher dose rate of 10 Gy/s of electron beam irradiation. Since the dose distributions in biological objects differ depending on the type of irradiation, which can have an impact on radio-biological effect, the research of electron beam irradiation at different doses and dose rates will allow insight into the mechanisms behind enzyme inactivation in biological objects, especially food products.

### 2.3. Catalase Activity in Non-Irradiated (Control) Solutions α at Different Concentrations

In our studies, special experiments were conducted using control model catalase solutions at different concentrations. These experiments were performed using both the spectrophotometric method and the oxygen bubble method. The results from these experiments served as the foundation for constructing the calibration curves (Section 2.3.3). This allowed us to evaluate the change in the concentrations of active catalase after e-beam irradiation at different doses.

#### 2.3.1. Catalase Activity in Non-Irradiated Solutions *α*: Spectrophotometry Method

**Figure 7:**
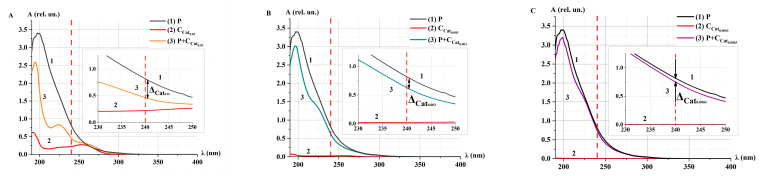
Absorption spectra of H_2_O_2_ (P, curve 1), catalase (Cat_i_, curve 2), and mixed solution (P+Cat_i_, curve 3) at different concentrations of catalase *i*. (**A**) Cat_0.03_, (**B**) Cat_0.003_, and (**C**) Cat_0.0003_. *t*_inc_ = 30 min; λ = 190–400 nm. The red dotted line marks the wavelength λ = 240 nm. The symbol ∆ and the black double headed arrow marks the difference in optical density between P and P+Cat_i_ at 240 nm.

Figure 7 shows the absorption spectra of P, Cat_i_, and P+Cat_i_ solutions, where *i* corresponds to the different concentrations of catalase in the model solutions. Each plot on the right side includes a zoomed-in scale for the wavelength range of λ = 230–250 nm.

Figure 7 shows that at *t*_inc_ = 30 min, the optical density of the P+Cat_i_ mixture at 240 nm decreases as the catalase concentration increases from 0.0003 to 0.03 units. The optical density of the solution changes in accordance with the concentration of substances [48]. The difference (∆) of the optical density of P and P+Cat_i_ solutions was smaller for solutions with lower catalase concentrations: ${∆}_{C_{0.03}}>{∆}_{C_{0.003}}>{∆}_{C_{0.0003}}$. This indicates slower decomposition of peroxide by the enzyme at lower enzyme concentrations, which aligns with data on the effects of different enzymes at varying concentrations [49,50].

**Figure 8:**
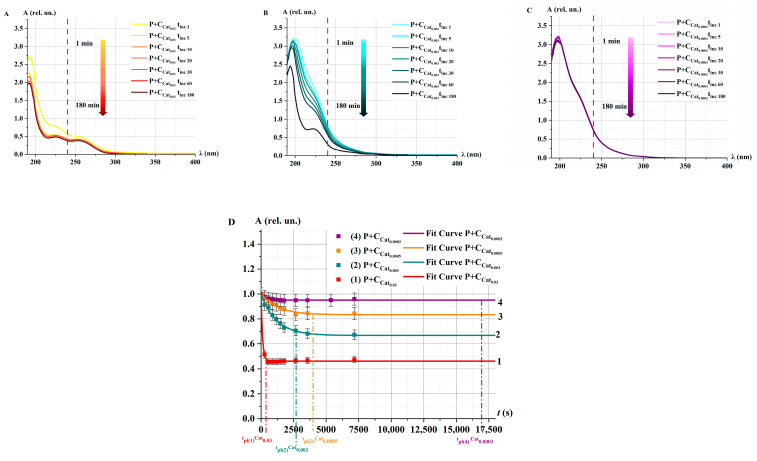
Absorption spectra of P+Cat_i_ solutions at different concentrations of catalase *i*: (**A**) Cat_0.03_, (**B**) Cat_0.003_, and (**C**) Cat_0.0003_ at *t*_inc_ = 1–180 min; λ = 190–400 nm. The red dotted line marks the wavelength λ = 240 nm. (**D**) Kinetic curves *A*(*t*); symbols represent experimental values (at λ = 240 nm). Fitting curves (Equation (1)) are represented by lines—Cat_0.03_ (curve 1), Cat_0.003_ (curve 2), Cat_0.0005_ (curve 3), and Cat_0.0003_ (curve 4). Normalization was performed on the value of optical density at *t*_inc_ = 0. The measurements are presented in the range *t*_inc_ = 0–18,000 s (5 h). The times of reaching the plateau $t_{pl(1)}$Cat_0.03_ = 370 ± 10 s for curve 1, $t_{pl(2)}$Cat_0.003_ = 2600 ± 50 s for curve 2, $t_{pl(3)}$Cat_0.0005_ = 4000 ± 60 s for curve 3, and $t_{pl(4)}$Cat_0.0003_ = 17,000 ± 100 s for curve 4, are indicated by dotted lines.

Figure 8 shows the kinetic optical density absorption spectra of the P+Cat_i_ solution at different catalase concentrations *i* for different *t*_inc_.

In the case of P+Cat_0.03_, an intense decrease in optical density was observed within the first 5 min after the addition of H_2_O_2_, and the absorption spectrum remained unchanged during further incubation (Figure 8A). For the Cat_0.003_ concentration, the decrease in the spectrum was much slower. Notably, in the case of P+Cat_0.0003_, almost no change in the absorption spectrum was observed (Figure 8B,C).

A nonlinear decrease in the optical density at 240 nm of the P+Cat_i_ solution was observed for different concentrations of the model catalase solution starting from *t*_inc_ (Figure 8D). The nature of the decrease in optical density at different concentrations corresponds well with the behavior of the kinetic curves observed for model solutions irradiated with accelerated electrons at different doses (Figure 3D). This suggests that the reduction in the rate of decline of the optical density of the P+Cat solution following e-beam irradiation may be related to a decrease in the concentration of active catalase.

#### 2.3.2. Catalase Activity in Non-Irradiated Solutions *α*: Oxygen Bubble Method

**Figure 9:**
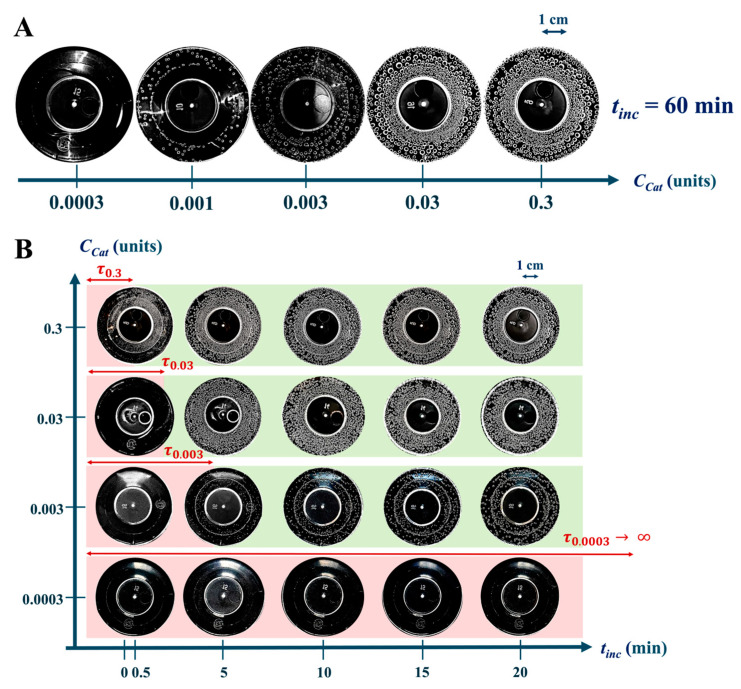
Photographs of oxygen bubbles in P+Cat_i_ solutions at different concentrations of Cat_0.0003_-Cat_0.3_ catalase. (**A**) *t*_inc_ = 60 min; (**B**) *t*_inc_ = 1–20 min. The time—when the bubbles were not identified—is highlighted in red, the time of their inception and development is highlighted in green.

Figure 9 shows photographs of P+Cat_i_ solutions at *i* from 0.0003 to 0.3 units, with oxygen bubbles formed on the surface of the solutions as a result of the catalytic reaction.

As the catalase concentration increased from Cat_0.0003_ to Cat_0.3_, a significant increase in the amount of oxygen released was observed (Figure 9A), specifically in the number of bubbles and their diameter at *t*_inc_ = 60 min. For the Cat_0.0003_ concentration, no bubbles were detected even after 60 min; i.e., the rate of catalytic reaction with oxygen generation at this enzyme concentration is approximately zero. On the contrary, for Cat_0.3_ concentration, the surface of the mixture solution was almost completely covered by a dense layer of bubbles after *t*_inc_ = 0.5 min, demonstrating the high activity of the enzyme at this concentration (Figure 9B). The red background highlights the period with no visible oxygen bubbles (lag time, τ), while the green background marks the onset of visually detectable oxygen release during the catalytic reaction. At all catalase concentrations, the number of oxygen bubbles increased over time, but the rate of this process depended on the enzyme concentration.

**Figure 10:**
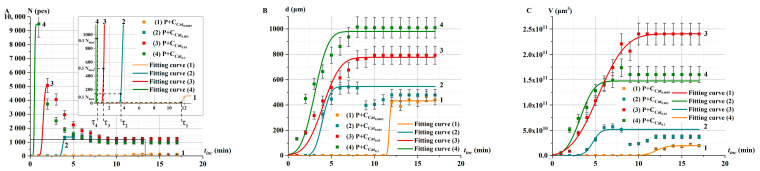
Kinetics of changes in oxygen bubble parameters in P+Cat_i_ solutions at different concentrations of model catalase solutions: Cat_0.0005_ (curve 1), Cat_0.003_ (curve 2), Cat_0.03_ (curve 3), and Cat_0.3_ (curve 4). (**A**) *N*(*t*_inc_), (**B**) *d*(*t*_inc_), and (**C**) *V*(*t*_inc_). Dots represent experimental values, and curves show fitting curve (Equation (3)). The dotted lines indicate the delay time τ.

Figure 10 shows the kinetics of changes in the number of *N*, *d*, and *V* in P+Cat_i_ solutions at different concentrations *i* of model catalase solutions.

With decreasing Cat_i_ concentration there was a decrease of bubble parameters *N*, *d*, and *V* from time *t*_inc_ (Figure 10A–C). The plots clearly show the delay times τ of bubble appearance. As Cat_i_ increases, the value of τ decreases. Similar to the case of e-beam irradiated solutions, after reaching the maximum value, the number of bubbles sharply decreases for high catalase concentrations Cat_0.3_–Cat_0.03_ due to merging and collapse (Figure 10A). In contrast, for low catalase concentrations Cat_0.003_–Cat_0.0005_, the number of bubbles remains almost unchanged throughout the incubation time. This further supports the idea that the mechanism of change in catalase activity after irradiation is related to a decrease in the concentration of initially active catalase.

#### 2.3.3. Calibration Curves for Doses

Experiments with different concentrations of catalase in control (non-irradiated) solutions *α* allowed us to establish a correlation between oxygen bubble parameters and spectral parameters. This correlation served as the basis for constructing calibration curves to determine the concentration of catalase in model solutions *β* irradiated with accelerated electrons.

Correlation coefficients were calculated between catalase concentrations (Cat_0.0001_–Cat_0.3_) and various parameters obtained by spectrophotometry method (*B_A_*, *k*, and *υ_A_*) and oxygen bubble method (τ, *B_F_*, *σ_F_*, and *t_F_*, where *F* = *N*, *d*, and *V*).

The parameters with the highest correlation coefficients (more than 0.98) were selected for constructing the calibration curves, indicating their high reliability. These parameters were the peroxide decomposition rate *υ_A_* (spectrophotometry method) and the inverse delay time 1/τ (oxygen bubble method). The calibration straight line was constructed using the following formula:

(4) $$y(C_{Cat})=aC_{Cat}+b,$$

where *a* is the slope of the straight line (tangent of the angle of inclination), and *b* is the *y*-intercept (the point where the line intersects the ordinate axis).

**Figure 11:**
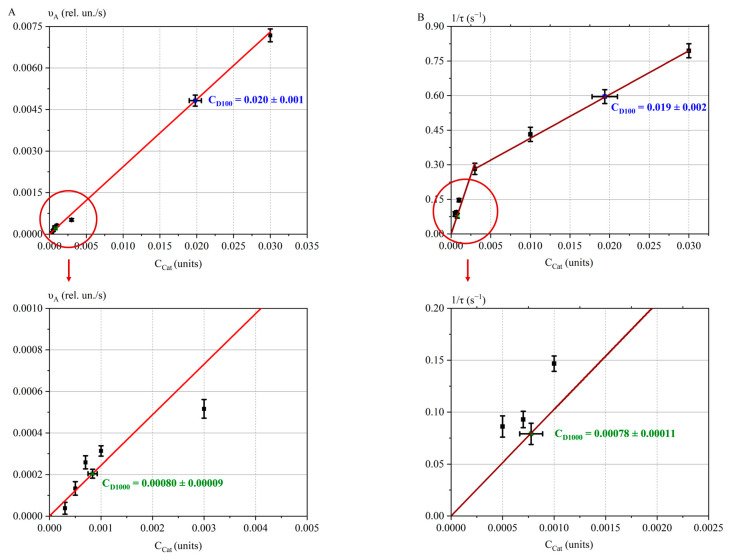
The experimental data (with error symbols) and calibration linear regressions: (**A**) *υ_A_*(*C*_Cat_)—red calibration line and (**B**) 1/τ(*C*_Cat_)—brown calibration line. Areas of study for small concentrations of Cat model solutions are highlighted in circles, with subsequent zooming in.

Figure 11 shows the parameters *υ_A_*(*C*_Cat_) and 1/τ(*C*_Cat_). The red straight lines represent the calibration curves, with experimental values for irradiated catalase solutions at doses D_100_ and D_1000_ plotted on them. The initial catalase concentration used was Cat_0.03_. This calibration was performed under the experimental conditions described in the Materials and Methods Section.

For the calibration curve of *υ_A_*(*C*_Cat_), the coefficients (Equation (4)) were determined as *a_υA_* = 0.24 ± 0.01 (rel. un./s) and *b_υA_* = (9.5 ± 0.2)∙10^−7^ (rel. un./s).

For the calibration curve of 1/τ(*C*_Cat_), the coefficients were *a*_1/τ_ = 92 ± 9 (rel. un./s) and *b*_1/τ_ = 0 ± 0 (1/s) for the concentration range Cat_0_–Cat_0.003_; *a*_1/τ_ = 18.9 ± 0.7 (rel. un./s) and *b*_1/τ_ = 0.234 ± 0.012 (1/s) for the concentration range Cat_0.003_–Cat_0.03_.

The irradiated catalase concentrations were estimated using the calibration straight lines: for the spectrophotometry method, *C*_D100_ = (0.020 ± 0.001) units and *C*_D1000_ = (0.00080 ± 0.00009) units, and for the oxygen bubble method, *C*_D100_ = (0.019 ± 0.002) units and *C*_D1000_ = (0.00078 ± 0.00011) units.

Thus, the same quantitative results were obtained using two independent methods, confirming the reliability of the quantitative assessment of changes in catalase activity after exposure to an accelerated electron beam. The results demonstrate that at a dose of 100 Gy, catalase activity decreased ~1.5-fold. When the dose was increased to 1000 Gy, the activity significantly decreased ~40-fold compared to the control samples.

It should be noted that the oxygen bubble method is a promising approach for the express diagnostics of catalase activity after exposure of biological systems to various physical and chemical factors. This method complements other techniques for assessing catalase activity based on the volume of oxygen released during the reaction [15,42].

The oxygen bubble method can be particularly useful in cases where the substrate spectrum overlaps with the P+Cat spectrum in spectrophotometric analysis. Such overlap may occur when working with solutions containing hemoglobin and myoglobin derivatives, as these compounds have their own absorption spectra in the 200–300 nm range, which can interfere with the results.

#### 2.3.4. Catalase-Based Biodosimeter for Measuring High Irradiation Doses

It was found that the most effective parameter for diagnosing catalase activity is the delay time τ—the duration of the time interval before active bubble release begins. Our experiments demonstrated that this time interval significantly depends on the dose of e-beam irradiation. This regularity can serve as the basis for developing a biodosimeter for the rapid assessment of high doses of irradiation.

The dependence of the inhibited catalase concentration on the irradiation dose follows an exponential behavior [8]. This is due to the probabilistic nature of the interaction between accelerated electrons and catalase molecules in model solutions, described by the following equation:

(5) $$C_{inh}(D)=C_{0}\left(1-e^{-\varphi D}\right),$$

where *C*_0_ is the initial concentration of catalase (in our experiments, 0.03 units), D (Gy) is the irradiation dose, and *φ* (Gy^−1^) is the catalase damage factor. Accordingly, the concentration of undamaged catalase *C*_Cat_ is described by the following equation:

(6) $$C_{Cat}(D)=C_{0}e^{-\varphi D}.$$

**Figure 12:**
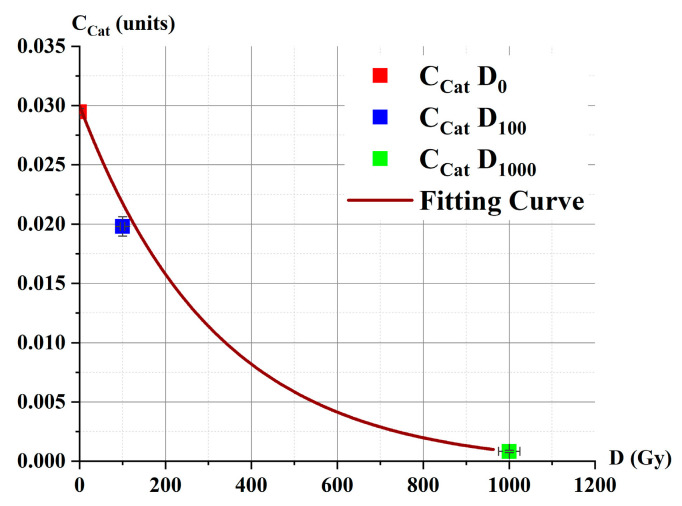
Dependence of the intact catalase concentration *C*_Cat_ as a function of irradiation dose D. Experimental points (squares). Parameters of fitting curve Equation (6) (brown line): *C*_0_ = 0.03 ± 0 units, *φ* = 0.0041 ± 0.0002 Gy^−1^, and correlation coefficient 0.98.

Figure 12 shows the experimental data for catalase concentrations (squares) obtained by irradiating model catalase solutions with accelerated electrons at doses D_0_, D_100_, and D_1000_. The fitting curve corresponding to these data, calculated using Equation (6), is also plotted.

**Figure 13:**
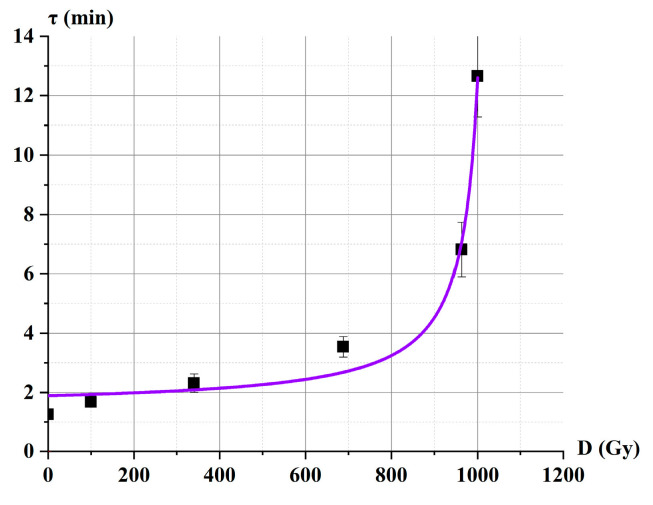
Dependence of the delay time τ on the irradiation dose D. Fitting curve parameters Equation (7) (purple line): τ_0_ = 1.89 ± 0.36 min, D_max_ = 1037 ± 13 Gy, *K* = 0.40 ± 0.13 min·Gy, and correlation coefficient 0.98.

Using the *C*_Cat_(D) dependence (Equation (6), Figure 12, brown line) and the 1/τ_i_(*C*_Cati_) calibration plot (Figure 11B), we derived the values for the τ_i_(D_i_) dependence, as shown in Figure 13.

To approximate the data, Equation (7) was chosen:

(7) $$\tau (D)=\tau_{0}+\frac{K\cdot D}{\left(D_{max}-D\right)},$$

where τ is the delay time for the irradiated solution; τ_0_ is the delay time for the catalase solution without irradiation D_0_; D_max_ is the dose at which *C*_Cat_ → 0, corresponding to complete inhibition of catalase in the solution; *K* and D_max_ are approximation coefficients.

The choice of Equation (7) is based on the observation that at a certain dose D → D_max_, catalase undergoes almost complete inhibition due to e-beam irradiation. The coefficient K characterizes the rate at which τ approaches infinity as D → D_max_. In other words, K reflects the sensitivity of the biodosimeter. This biodosimeter, with an initial catalase concentration of Cat_0.03_, can be used to measure doses in the range of 400–1000 Gy. To extend the range of measurable doses, additional studies are required.

The clear dose dependencies of the time before oxygen release begins and the hydrogen peroxide decomposition rate in catalase saline solutions suggest that in real life, food products’ catalase activity depends on irradiation dose. According to recent studies [15,46] proving that catalase activity decreases with an increase in the irradiation dose exceeding 100 Gy and as found also in our study, it can be assumed that catalase activity in food products would decrease with an increase in the irradiation dose [51,52,53]. Since enzymes, especially catalase, extend the shelf life of food products by decomposing ROS occurring in foods during long-term storage, food irradiation should be performed to minimize the damage to the enzymes. Measuring catalase activity in food products after irradiation with different doses would allow to determine the optimal dose range bearing in mind individual properties of different categories of food products as well as the individual compositions of antioxidants. Being easily applicable to diverse animal and plant products, the oxygen bubble method described in the study can be used as an express method for determining the precise irradiation dose limit that should not be exceeded, as a higher dose would cause damage to the enzymes.

## 3. Materials and Methods

All stages of the experiment are presented in Figure 1.

### 3.1. Objects of Study and Sample Preparation

The reagents used in the study included catalase enzyme solution (activity: 5000 units/cm^3^, batch № 08-22, LLC “EdaProf”, Moscow, Russia, referred to as stock solution) and a hydrogen peroxide solution (3% H_2_O_2_; LLC “Samara Pharmaceutical Factory”, Samara, Russia). All reagents were stored in their required temperature conditions: catalase at 3–4 °C and hydrogen peroxide at room temperature.

The catalase enzyme solution was diluted in distilled water to prepare the basic solution at different concentrations (Stage 1 in Figure 1). The concentrations of the basic solutions were as follows: 0.0001 units (0.05 µL Cat, 50 mL water), 0.0003 units (0.15 μL Cat, 50 mL water), 0.0005 units (0.25 µL Cat, 50 mL water), 0.001 units (0.5 µL Cat, 50 mL water), 0.003 units (1.5 µL Cat, 50 mL water), 0.01 units (5 µL Cat, 50 mL water), 0.03 units (15 µL Cat, 50 mL water), and 0.3 units (150 µL Cat, 50 mL water). All catalase solutions were prepared immediately prior to irradiation.

### 3.2. Experiment with Different Concentrations of Catalase

To construct the calibration curve for catalase activity, a model experiment was conducted using different concentrations of catalase solutions (Stage 2 in Figure 1). For this purpose, the basic solution was diluted to various concentrations, and 5 mL of each solution was placed in plastic containers with lids (diameter: 48 mm, volume: 120 mL, LLC “PLASTILAB INDUSTRY”, Saint Petersburg, Russia) and referred to as solution *α*. Next, 83 µL of 3% (*w*/*v*) H_2_O_2_ was added to solution *α* to initiate the catalytic reaction. Subsequently, spectrophotometric measurements and oxygen bubble measurements were performed to determine catalase activity. All experiments were conducted at a room temperature of 20 °C.

### 3.3. e-Beam Irradiation

The samples of catalase solutions were irradiated using a continuous electron accelerator UELR-1-25-T-001 (Skobeltsyn Institute of Nuclear Physics at Moscow State University, Moscow, Russia), with a maximum energy of 1 MeV and an average beam power of 25 kW (Stage 3 in Figure 1).

Six plastic jars with solution *α* samples were put on a 35 cm × 5.2 cm duralumin plate. The catalase solutions were irradiated in three separate irradiation sessions for each dose. The height of solution *α* in the jars did not exceed 3 mm, and a volume of 5 mL was selected to ensure uniform irradiation. This volume was chosen due to the limited penetration depth of 1 MeV electrons, which is no more than 5 mm.

During irradiation, the charge Q_exp_ absorbed by the part of the duralumin plate not occupied by the samples was recorded [8]. This charge was used to determine the dose absorbed by the samples using an analog-to-digital converter (OOO Industrial Association “Oven”, Moscow, Russia). The margin of error in determining the charge was no more than 2% [54].

The irradiation doses were (100 ± 5) Gy and (1000 ± 25) Gy, with a dose rate of 10 Gy/s. The ambient temperature during irradiation was 20 °C, and the control solutions *α* were stored under the same temperature conditions as the irradiated solutions *β*.

Five minutes after irradiation, 83 μL of 3% H_2_O_2_ was added to the *α* and *β* catalase model solutions to initiate the reaction. After a certain period of time (*t*_inc_), the solutions were then analyzed spectrophotometrically, and the oxygen yield was determined.

### 3.4. Spectrophotometry Measurement

The catalase activity was assessed using the spectrophotometric method (Stage 4 in Figure 1). After adding hydrogen peroxide to solutions *α* and *β*, the solutions were placed in Ultra 10 mm quartz cuvettes (12.5 × 12.5 × 45 mm^3^, KU-10.10 A, Ultra Optic Cell Co., Ltd., Saint Petersburg, Russia). The solutions were stirred, and after 2–5 s, the absorption spectra were measured using a UV-3000 spectrophotometer (Shanghai Mapada Instruments Co., Ltd., Shanghai, China) in the wavelength range of 190 to 400 nm.

For the model experiment with varying catalase concentrations, the spectra of solutions *α* and *β* were measured every 30 s for the first 3 min, then every 2 min for the next 30 min, and finally at 60, 90, 120, 150, and 180 min. The catalase activity was determined from the hydrogen peroxide decomposition rate *υ_A_* (Equation (2)), which is proportional to absorbance coefficient at a wavelength of λ = 240 nm.

### 3.5. Oxygen Bubble Measurement

In our experiments, the activity of catalase was also evaluated using the oxygen bubble method (Stage 5 in Figure 1). This method involves measuring the oxygen bubbles formed in the catalase solution following the addition of hydrogen peroxide in the catalytic reaction $2H_{2}O_{2}\overset{Cat}{\to }2H_{2}O+O_{2}$.

After adding H_2_O_2_ to solutions α and β, the solutions were thoroughly mixed. Photographs of the monolayer of bubbles at the bottom of the container were taken using a camera phone. For both the model experiment and the irradiation experiment, photographs were captured every 30 s for 60 min.

The photographs were then processed using ImageJ 1.54j software (National Institutes of Health, Bethesda, MD, USA). The number of bubbles (*N*), their average diameter (*d*), and the volume (*V*) of all bubbles were calculated.

### 3.6. Construction of Calibration Curves

Experiments were conducted using different model solutions of catalase at varying concentrations Cat_i_ to construct the calibration curves. The parameters selected for constructing the calibration curves were chosen based on a high correlation coefficient (greater than 0.98) between the parameter and the *C*_Cat_.

### 3.7. Statistical Analysis of the Data

Model solutions of catalase were irradiated with accelerated electrons three times at each dose. For each non-irradiated solution *α* and irradiated solution *β*, the absorption spectra were measured three times, and the oxygen bubble indices were also measured three times.

Statistical processing and the plotting of all approximating curves were performed using the Origin Pro 2024 program (OriginLab Corporation, Northampton, MA, USA). All graphs present experimental data in means ± SD format.

Correlation coefficients were calculated for the parameters measured using both the spectrophotometric and oxygen bubble methods.

## 4. Conclusions

The present study performed to assess the change in catalase activity in saline solution irradiated with 1 MeV accelerated electrons showed that e-beam irradiation with the doses of 100 Gy and 1000 Gy at the dose rate of 10 Gy/s decreases catalase activity, which manifests in a lower hydrogen peroxide decomposition rate *υ_A_*(*C*_Cat_), measured using spectrophotometry method, and a delayed appearance of oxygen bubbles 1/τ(*C*_Cat_) after adding hydrogen peroxide to catalase saline solution. The difference between catalase activity estimated using the spectrophotometry method and the express oxygen bubble method developed by our team was not more than 5%, which attests to the adequacy of the proposed methodology. The calibration dependencies of hydrogen peroxide decomposition rate and the bubble inverse delay time on the catalase concentration observed in model experiments with different concentrations of catalase were chosen to quantify the concentration of active catalase after e-beam irradiation with different doses. The calibration dependencies showed that while low-energy electrons with the dose of 100 Gy decreased the catalase activity approximately 1.5 times compared to the activity of non-irradiated catalase in saline solution, the dose of 1000 Gy reduced the activity of catalase approximately 40 times. Since the catalase activity decreases with an increase in the irradiation dose, it can serve as a quantifiable marker of the damage to proteins and enzymes in food products during irradiation.

The obtained dose dependencies of the hydrogen peroxide decomposition rate and the bubble inverse delay time on the irradiation dose allowed us to construct the dose–response dependency of the delayed appearance of oxygen bubbles τ, which can be used as a basis for the development of a biodosimeter for the quick assessment of radiation exposure. This study demonstrates the potential of the visual oxygen bubble method for express diagnostics of the catalase activity change triggered by various physical and chemical impacts on diverse biological objects.

This study of the changes in catalase activity after irradiation, which we performed using catalase solutions, can be extended to research antioxidant properties of diverse food products containing various antioxidants after irradiation for developing reliable markers for detection of the fact of irradiation and overexposure of food products during long-term storage. Our further studies will extend the catalase research on model solutions to irradiated animal and plant products to establish precise criteria for choosing optimal parameters for food irradiation.

## Data Availability

The original contributions presented in the study are included in the article; further inquiries can be directed to the corresponding author/s.
